# Pdcd4 Is Involved in the Formation of Stress Granule in Response to Oxidized Low-Density Lipoprotein or High-Fat Diet

**DOI:** 10.1371/journal.pone.0159568

**Published:** 2016-07-25

**Authors:** Yang Bai, Zhaojing Dong, Qianwen Shang, Hui Zhao, Liyang Wang, Chun Guo, Fei Gao, Lining Zhang, Qun Wang

**Affiliations:** 1 Department of Immunology, Shandong University School of Medicine, Jinan 250012, Shandong, China; 2 The Key Laboratory of Cardiovascular Remodeling and Function Research, Chinese Ministry of Education and Chinese Ministry of Public Health, Qilu Hospital, Shandong University, Jinan 250012, Shandong, China; Laval University, CANADA

## Abstract

Stress granules (SGs) in response to various stresses have been reported in many diseases. We previously reported the implication of programmed cell death 4 (Pdcd4) in obesity-induced stress responses, but the possible link between Pdcd4 and SGs remains lacking. In this study we showed that oxidized low-density lipoprotein (ox-LDL) or high-fat diet (HFD) induced SG formation in mouse macrophages and liver tissues, and Pdcd4 deficiency in mice remarkably reduced its formation. In response to ox-LDL, either endogenous or ectopic Pdcd4 displayed granule-like expression and co-localized with SG markers including T-cell-restricted intracellular antigen-1, fragile X mental retardation-related protein 1, and eukaryotic initiation factor 4A. Ectopic expression of truncated Pdcd4 that depleted specific RNA-binding motif significantly disrupted the SG formation, suggesting the direct involvement of Pdcd4 in ox-LDL-induced SGs through its RNA-binding activity. Additionally, Pdcd4 deficiency drove AKT activation and suppression of eIF2α phosphorylation, thereby contributing to the resistance to ox-LDL or HFD-induced SG formation. Collectively, our data suggest that Pdcd4 as a crucial regulator in SGs induced by ox-LDL or HFD maybe a potential target for mitigating SG-associated stress responses in obesity and related diseases.

## Introduction

Stress granules (SGs) are translationally silent cytoplasmic ribonucleoprotein complexes that assemble during various types of cellular stresses such as heat shock, viral infection, oxidative and/or endoplasmic reticulum (ER) stresses [1–3]. In response to environmental emergency, cells can trigger a sudden translational arrest, leading to rapid polysome disassembly and subsequent assembly of heterogeneous mRNAs and translation factors[4]. Although the composition can vary depending on various stimuli, SGs typically contain mRNA, 40S ribosomal subunits, eukaryotic translation initiation factor (eIF) 4E, eIF4G, eIF4A, eIF4B, poly(A)-binding protein, eIF3 and eIF2, other RNA-binding proteins including T-cell-restricted intracellular antigen-1 (TIA-1), TIA-1-related RNA protein (TIAR), fragile X mental retardation-related protein 1 (FXR1), and Ras-GAP SH3 domain-binding protein are also included in the assembly of SGs [1, 2].

So far, SGs have been implicated in various disease conditions including viral infection, inflammatory diseases, cancer and multiple neurodegenerative diseases, in which they may exert either beneficial or adverse effects [2, 5–7]. Therefore, the regulation of SG assembly is important under different pathological conditions. Stress-induced phosphorylation of eIF2α is required for SG assembly in many stress responses through inhibiting cap-dependent translation initiation [1, 2]. AKT, also known as protein kinase B, that regulates cell survival, growth, metabolism and stress [8, 9], has recently been reported to suppress eIF2α phosphorylation and contribute to relieved ER or oxidative stress [10]. However, the direct link between AKT and eIF2α during SG assembly remain unestablished.

Programmed cell death 4 (Pdcd4), initially identified as an up-regulated gene during apoptosis, is expressed widely in normal tissues. It has been well documented that Pdcd4 acts as a regulator of gene expression through influencing translation and transcription. Pdcd4 selectively inhibits cap-dependent translation through binding to RNA helicase eIF4A with its MA-3 domains, which is highly homologous to eIF4G [11–13]. It has been well recognized that Pdcd4 inhibits neoplastic transformation and tumor progression through its inhibitory role in protein translation [12, 14]. Previous studies have reported the pro-inflammatory role of Pdcd4 through suppressing the production of immunomodulatory cytokine IL-10 [15, 16]. Recently, we have demonstrated the involvement of Pdcd4 in obesity, adipose inflammation, and atherosclerosis [13, 17]. Pdcd4 deficiency leads to an obvious alleviation in high-fat diet (HFD)-induced adipose ER stress and hepatic oxidative stress, which are typical hallmarks of both obesity and cardiovascular events and link with inflammation on different levels [13, 18–21]. These findings suggest that Pdcd4 plays a critical role during HFD-induced stress processes in the pathogenesis of obesity and related diseases. Despite the important role of Pdcd4 during lipid disorders [13], direct evidence for the involvement of Pdcd4 in these stress processes remains lacking. The possible link between Pdcd4 and SGs in response to HFD factors is yet to be determined.

As an inducer of SGs, oxidative stress is persistently present in HFD-induced obesity and metabolic syndromes. A typical biomarker for oxidative stress in these pathological conditions is oxidized low-density lipoprotein (ox-LDL), which contributes to the enhanced oxidative stress in macrophages and some other cells through promoting and augmenting the generation of reactive oxygen species [20–28]. In the present study, we show that either ox-LDL or HFD serves as stress signal to induce the formation of SGs in macrophages and/or liver tissues. During ox-LDL or HFD-induced SGs, Pdcd4 functions as a critical regulator, which not only directly participates in the assembly of SGs through its RNA-binding region, but also regulates SG formation probably through AKT-eIF-2α axis. Thus, we provide a potential link between HFD-associated SGs and the pathogenesis of obesity and related diseases, indicating that Pdcd4 may be a novel therapeutic target for resolving SG-associated stress responses.

## Materials and Methods

### Animals

Wild-type (WT) and Pdcd4-deficient (*Pdcd4*^*-/-*^) male mice on a C57BL/6 background were used in this study. *Pdcd4*^*-/-*^ mice were kindly provided by Youhai H. Chen, University of Pennsylvania School of Medicine. In some experiments, mice were fed on HFD containing 15% w/w fat and 0.25% cholesterol to induce obesity. This study was performed in strict accordance with the institutional guidelines for animal care and utilization. The protocol was approved by Ethical Review Committee of Animal Experiments of Shandong University School of Medicine (Permit Number: NO.ECAESDUSM 2012033). Mice were sacrificed under anesthesia, and all efforts were made to minimize mice suffering.

### Cell culture

Primary peritoneal macrophages were harvested from peritoneal exudates of mice, which were injected intraperitoneally with 6% hydroxyethyl starch solution 3 days before the harvest. After 6 h of incubation, non-adherent cells were removed and the adherent cells were used as peritoneal macrophages. HeLa (human cervical carcinoma) and HepG2 (human hepatoblastoma) cell lines were obtained from Shanghai Cell Bank of Chinese Academy of Sciences (Shanghai, China), and maintained at 37°C under 5% CO_2_ in Dulbecco’s modified Eagle’s medium containing 10% fetal bovine serum (Invitrogen, Carlsbad, CA), 100 U/ml penicillin, and 100 mg/ml streptomycin.

### Cell treatment

Primary macrophages from WT or *Pdcd4*^*-/-*^ mice were treated with ox-LDL (50μg/ml, Yiyuan, Beijing, China) for 24 h, and then were used to perform western-blot or immunofluorescence. In some experiments, AKT inhibitor MK2206 (Selleck, Houston, TX) was added 6 h before the addition of ox-LDL. For HeLa or HepG2 cells, ox-LDL (50μg/ml) treatment for 24 h or 16 h was performed after overnight transfection with different plasmids.

### Plasmid construct and transfection

*pEGFP-C1* or *pEGFP-C1-Pdcd4* plasmids were constructed and kindly provided by Olubunmi Afonja, New York University. As referring to the study by Klempnauer et al [29], truncated plasmids were constructed based on *pEGFP-C1-Pdcd4* plasmid by depletion of RNA binding motif (RBM)1 sequence from 151 to 204 bases (*pEGFP-C1-Pdcd4-D1*), RBM2 sequence from 289 to 390 bases (*pEGFP-C1-Pdcd4-D2)*, and both RBM1 and RBM2 sequences (*pEGFP-C1-Pdcd4-D1+2*), respectively. HeLa or HepG2 cells were transfected with the above plasmids using the Lipofectamine 2000 transfection reagent (Invitrogen, Carlsbad, CA) as per the manufacturer’s instructions.

### Immunofluorescence

Cells planted in 24-well chamber slides were fixed in 4% paraformaldehyde for 30 mins and blocked with 5% Bovine serum albumin (BSA) for 1 h, and then the cells were incubated with primary antibodies (Abs) against Pdcd4 (1:300, #9535, Cell Signaling Technology, Beverly, MA), TIA-1(1:100, sc-1751), FXR1(1:100, sc-10554), or eIF4A(1:100, sc-14211) (Santa Cruz, Dallas, TX) at 4°C, overnight. After washing, the cells were incubated with fluorescein isothiocyanate (FITC)-conjugated or Rhodamine-conjugated secondary Abs (1:300, SA00003-8, SA00007-3) (Proteintech Group, Chicago, IL). The cell nuclei were stained with 4, 6-diamidino-2-phenylindole (DAPI) (Beyotime Biotechnology, Shanghai, China). Immunofluorescence signals were visualized with fluorescence microscope (Axio Imager A2) or laser scanning confocal microscope (LSM780) (Zeiss, Jena, Germany). To detect the formation of SGs in liver tissues from ND or HFD-fed WT or *Pdcd4*^*-/-*^ mice, paraffin sections were stained with anti-TIA-1 Ab followed by Rhodamine-conjugated secondary Ab. Nuclei were stained by DAPI.

### Western-blot assay

Cells were lysed in protein lysis buffer in the presence of protease inhibitor and phosphatase inhibitor. Equal amounts of protein were separated on SDS-PAGE and transferred onto polyvinylidenefluoride membranes (Millipore, Billerica, MA). After blocking with 5% BSA in TBST containing 0.1% Tween-20 for 3 h, the membrane was incubated overnight at 4°C with primary Abs against Pdcd4 (1:1000, #9535), p-eIF2α (1:1000, #3597), eIF2α (1:1000, #5324), p-AKT (1:2000, #4060) (Cell Signaling Technology, Beverly, MA), AKT (1:1000, #1081–1) (Epitomics, Burlingame, CA), followed by peroxidase-labeled secondary Abs for 1 h at room temperature. After washing, signals were visualized by SuperSignal West Pico Chemiluminescent Substrate (Pierce Biotechnology, Rockford, IL).

### Statistical analysis

Statistical analysis was performed using one-way ANOVA or student-*t* test (GraphPad Prism 5). Data are presented as the mean ± s.e.m. Statistical significance was established at *P* values< 0.05.

## Results

### Pdcd4 deficiency reduces the SG formation in macrophages and liver tissues from HFD-fed mice

**Fig 1:**
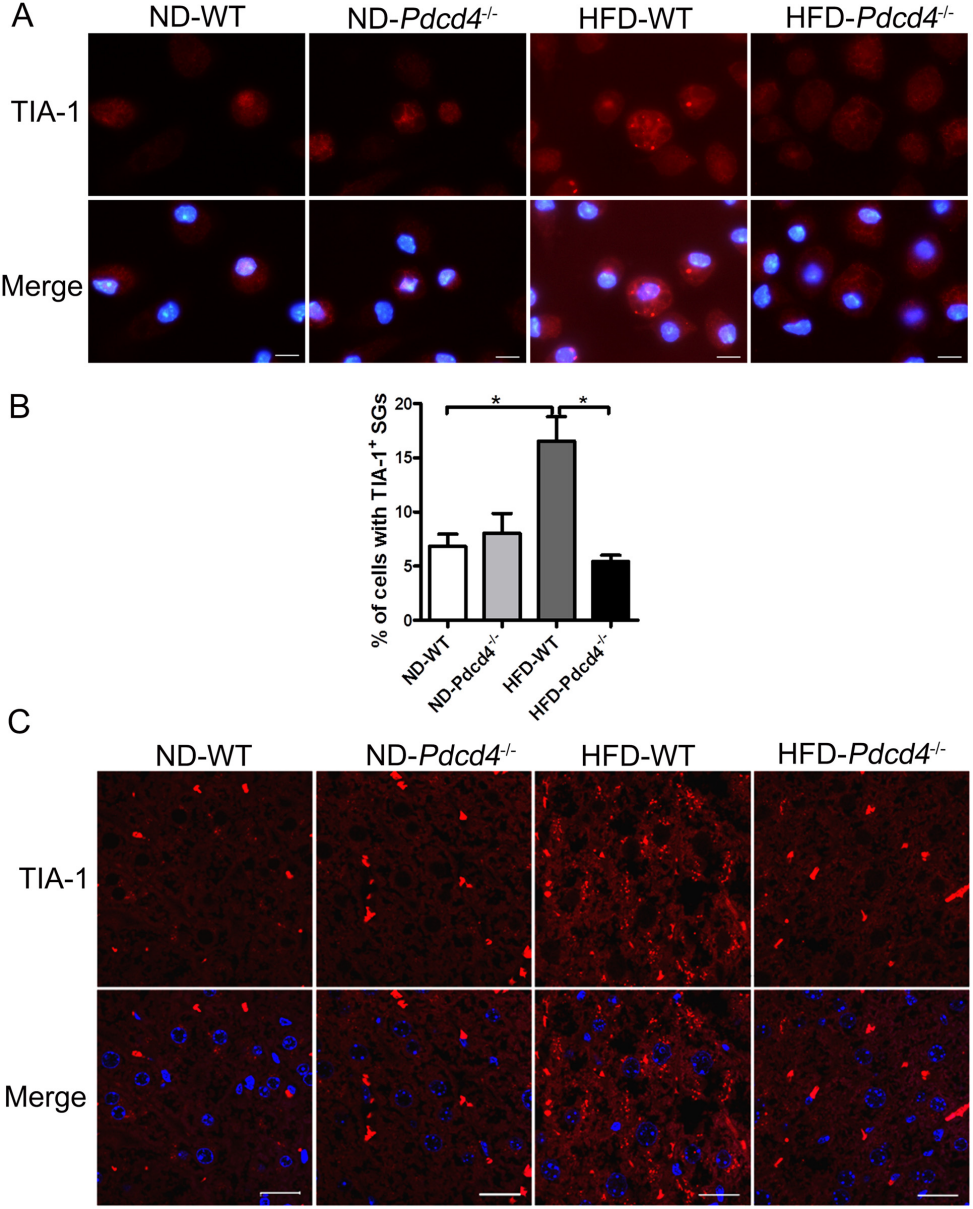
Pdcd4 deficiency reduces the SG formation in macrophages and liver tissues from HFD-fed mice. (A, B) The formation of TIA-1^+^ SGs was examined in primary macrophages from WT and *Pdcd4*^*-/-*^ mice (n = 2–4 per group) fed on ND or HFD by immunofluorescence. Representative (A) and statistical (B) data are shown. *P <0.05. (C) The formation of TIA-1^+^ SGs was examined in liver tissues from WT and *Pdcd4*^*-/-*^ mice (n = 4 per group) fed on ND or HFD by immunofluorescence. TIA-1 (red); nuclei (blue). The original magnification is 1000 (A) or 630 (C). Scale bar = 10μm.

Pdcd4 has been demonstrated to be involved in HFD-induced obesity and associated stress responses [13]. To determine the possible link between Pdcd4 and SGs upon HFD challenge, we detected the formation of SGs in macrophages and liver tissues in established HFD-fed WT and *Pdcd4*^*-/-*^ mice models. Based on our previous study, although WT and *Pdcd4*^*-/-*^ mice were both fed on HFD, WT mice developed obesity, while *Pdcd4*^*-/-*^ mice displayed lean phenotype [13]. Using TIA-1 as a SG marker, we found that about 16.5% of WT macrophages isolated from HFD-fed mice displayed TIA-1 positive SGs, a significant increase when compared to macrophage isolated from ND-fed mice (about 6.8%). This result suggests that indeed, HFD induces a stress response in WT macrophages. However, macrophages isolated from *Pdcd4*^*-/-*^ mice on HFD did not display this increase, showing that Pdcd4 is required for SG formation under HFD (Fig 1A and 1B). Similar effects were observed in liver sections from WT and *Pdcd4*^*-/-*^ mice. HFD-fed WT mice showed conspicuous hepatic steatosis and much more cells displayed SGs when compared to mice fed on ND (Fig 1C). These data indicate that HFD induces the formation of SGs, and importantly, reveal that Pdcd4 serves as key contributor to SG formation induced by HFD stimulus.

### Pdcd4 deficiency reduces the formation of TIA-1^+^ SGs in macrophages in response to ox-LDL

**Fig 2:**
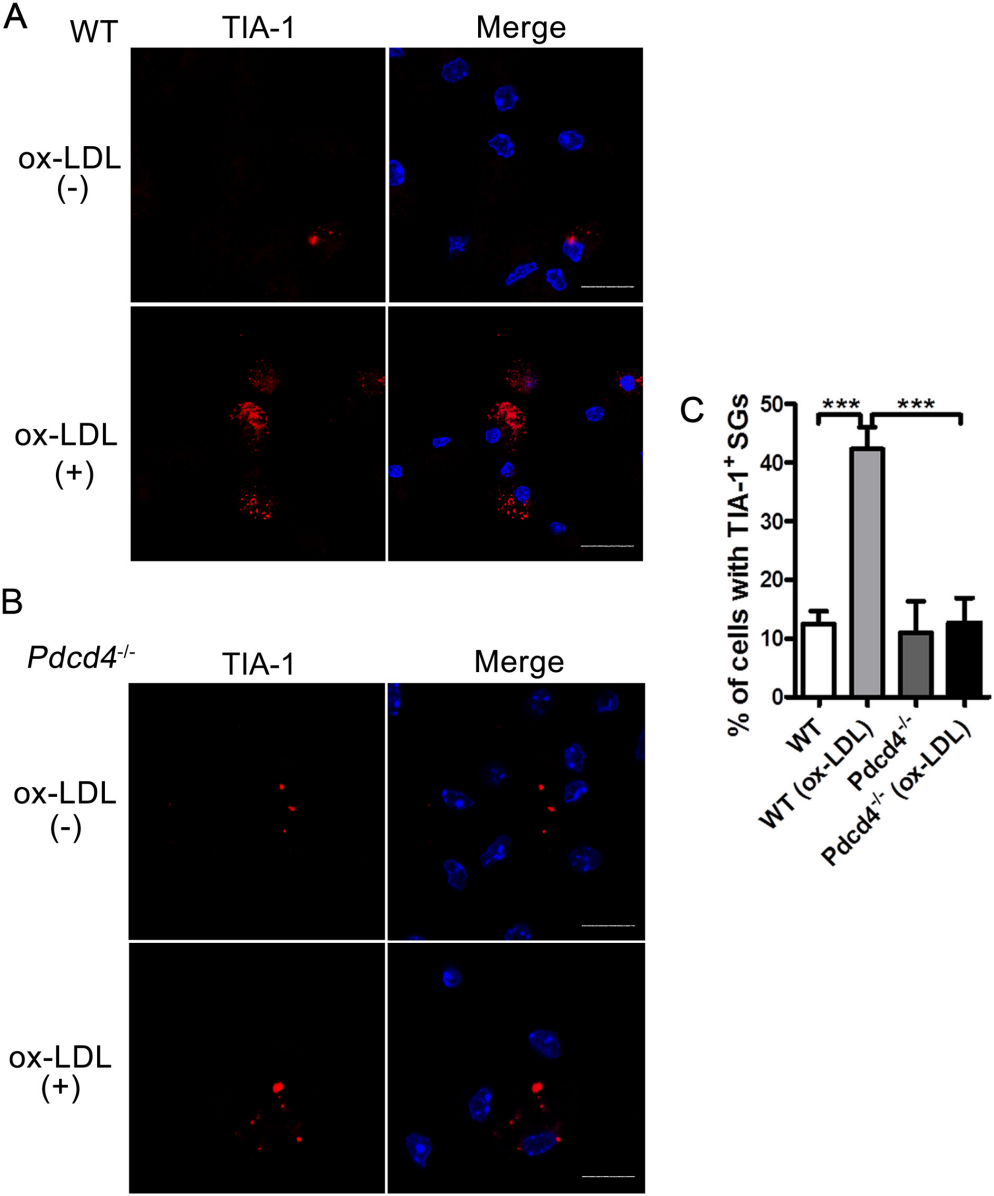
Pdcd4 deficiency reduces the formation of TIA-1^+^ SGs in ox-LDL-treated macrophages. (A, B) Primary macrophages from WT (A) or *Pdcd4*^-/-^ (B) mice (n≥3 per group) were stimulated with ox-LDL (50 μg/ml) for 24 h, the formation of SGs was examined by indirect immunofluorescence. TIA-1 (red); nuclei (blue). The original magnification is 630. Scale bar = 10 μm. (C) Positive percentage of cells containing TIA-1^+^ SGs. Data are presented as mean ± s.e.m. ***P <0.001.

Since ox-LDL is a typical biomarker for oxidative stress in HFD-induced obesity and related metabolic syndromes, we next used ox-LDL to treat primary peritoneal macrophages from WT and *Pdcd4*^*-/-*^ mice, and examined TIA-1^+^ SGs in these macrophages. In response to ox-LDL, the TIA-1^+^ SGs in WT macrophages significantly increased as suggested by elevated percentage of cells containing TIA-1^+^ SGs compared with non-treated macrophages (Fig 2A and 2C). However, *Pdcd4*^*-/-*^ macrophages treated with ox-LDL did not show this increase (Fig 2B and 2C), showing that Pdcd4 is required for SG formation under ox-LDL. The SGs were only observed in a few of *Pdcd4*^*-/-*^ macrophages, which is similar to non-treated macrophages (Fig 2B and 2C). These results suggest that Pdcd4 deficiency causes reduced SG formation in response to ox-LDL, thus revealing a key role of Pdcd4 in ox-LDL-induced SGs.

### Pdcd4 is co-localized with specific markers of SGs in ox-LDL-stimulated macrophages

**Fig 3:**
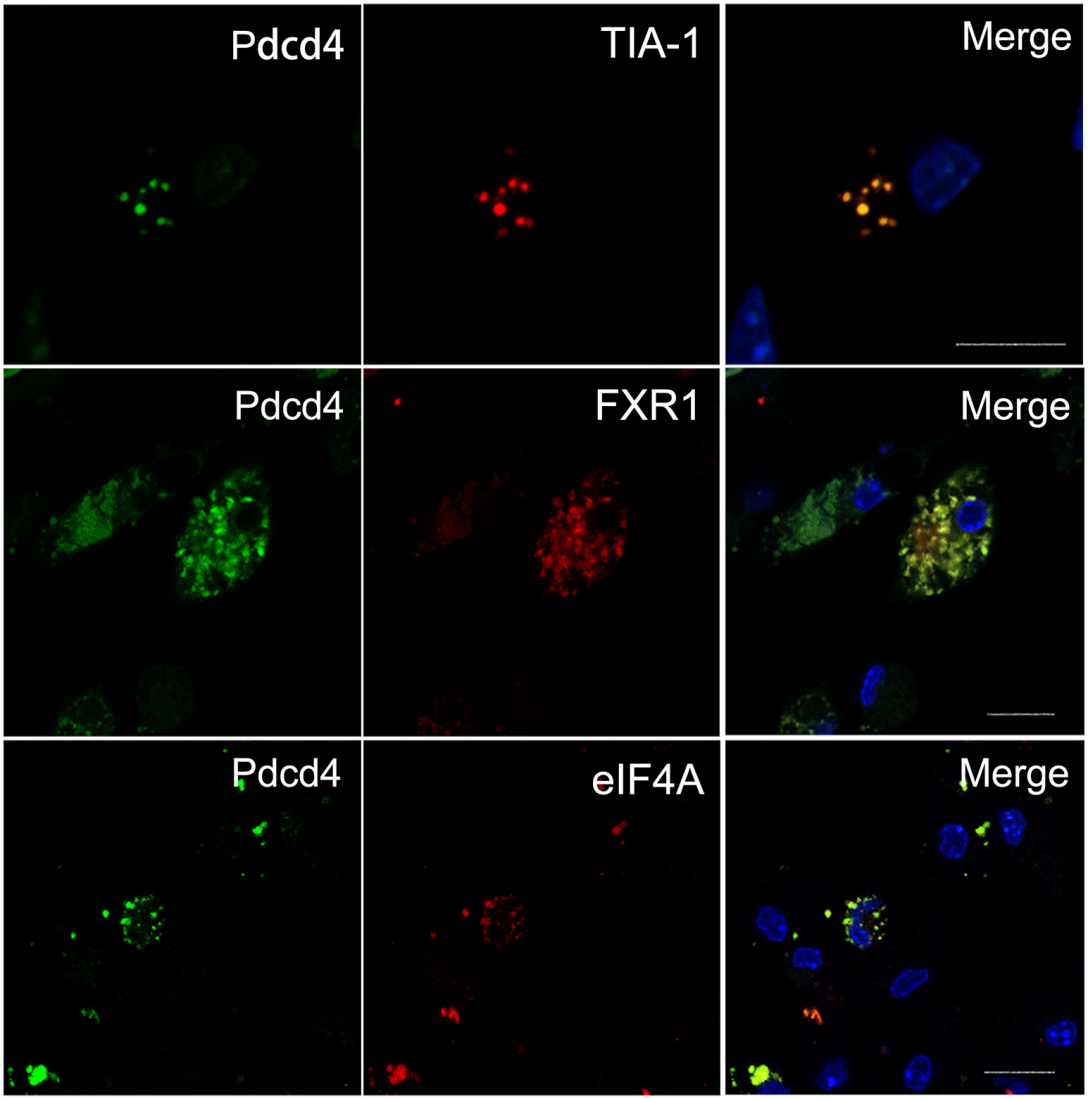
Pdcd4 is co-localized with specific markers of SGs in ox-LDL-treated macrophages. Primary macrophages from WT mice (n = 6) were stimulated with ox-LDL (50 μg/ml) for 24 h. The expression and co-localization of Pdcd4 and SG markers were examined by immunofluorescence. Pdcd4 immunoreactivity was visualized with FITC (green), TIA-1, FXR1 and eIF4A were detected with rhodamine (red). Cell nuclei were stained with DAPI (blue). The original magnification is 630. Scale bar = 10 μm.

To verify the role of Pdcd4 in ox-LDL-induced SGs, the expression of Pdcd4 and other related SG marker proteins were examined in macrophages by immunofluorescence. Fig 3 showed that in response to ox-LDL, Pdcd4 clearly displayed granule-like expression in the perinuclear area of the macrophages and all the detected SG markers including TIA-1, FXR1 and eIF4A were positively expressed. Notably, these markers apparently co-localized with Pdcd4 in the fluorescence granules. While in non-treated macrophages, the expression pattern of TIA-1, FXR1 and eIF4A exhibited rare fluorescence granules (S1 Fig). These data suggest that ox-LDL can induce SG formation in the primary macrophages, and Pdcd4 may be an important component of ox-LDL-induced SGs.

### Ectopic Pdcd4 is co-localized with specific markers of SGs in ox-LDL-stimulated HeLa cells

**Fig 4:**
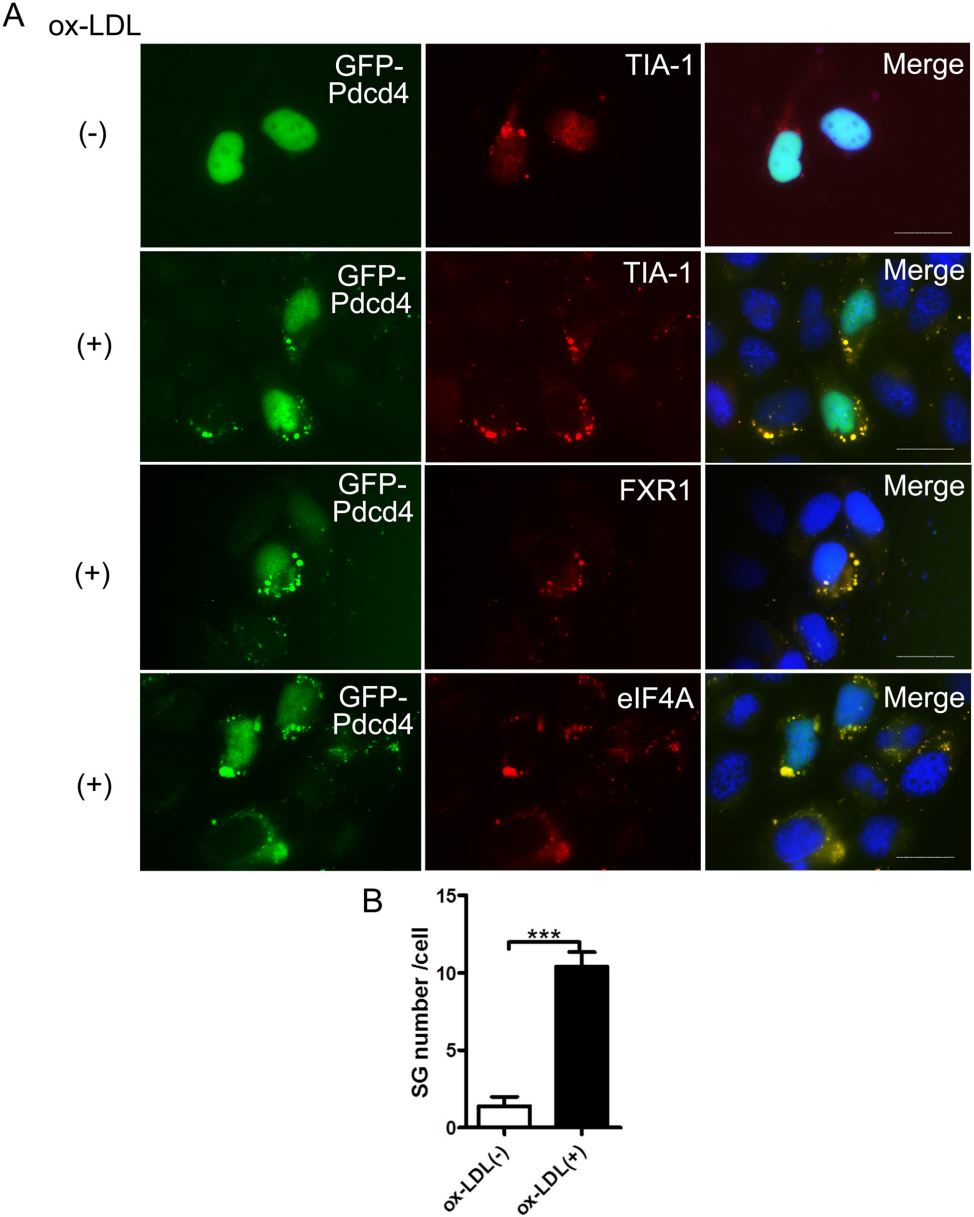
Ectopic Pdcd4 is co-localized with specific markers of SGs in ox-LDL-treated HeLa cells. HeLa cells were transfected with *pEGFP-C1-Pdcd4* in the presence or absence of ox-LDL (50 μg/ml) for 24 h. Intracellular localization of exogenous Pdcd4 was visualized with GFP (green). SG markers (TIA-1, FXR1 and eIF4A) were examined by rhodamine (red) using immunofluorescence. Cells were counterstained with DAPI (blue). The original magnification is 1000. Scale bar = 20 μm. Representative (A) and statistical (B) data are shown. Data represent more than three independent experiments with similar results. ***P <0.001.

To confirm the involvement of Pdcd4 in ox-LDL-induced SGs, HeLa cells were transfected with *pEGFP-C1-Pdcd4* plasmid, and then were treated with ox-LDL, the formation of SGs were determined. In non-treated HeLa cells, exogenous GFP-fused Pdcd4 was mostly expressed in nuclei and only a few SGs were detected. While in ox-LDL-treated HeLa cells, exogenous Pdcd4 was expressed in both nuclear and cytoplasmic location, and obvious SGs were observed. Of note, the cytoplasmic Pdcd4 displaying granule-like expression was also co-localized with SG markers including TIA-1, FXR1 and eIF4A (Fig 4A and 4B). These data are partially in consistent with the observed finding in macrophages, thereby suggesting that ox-LDL treatment also drives exogenous Pdcd4 into SG assembly and further confirming the involvement of Pdcd4 in ox-LDL-induced SGs.

### Pdcd4 participates in the formation of SGs through its RNA-binding region

**Fig 5:**
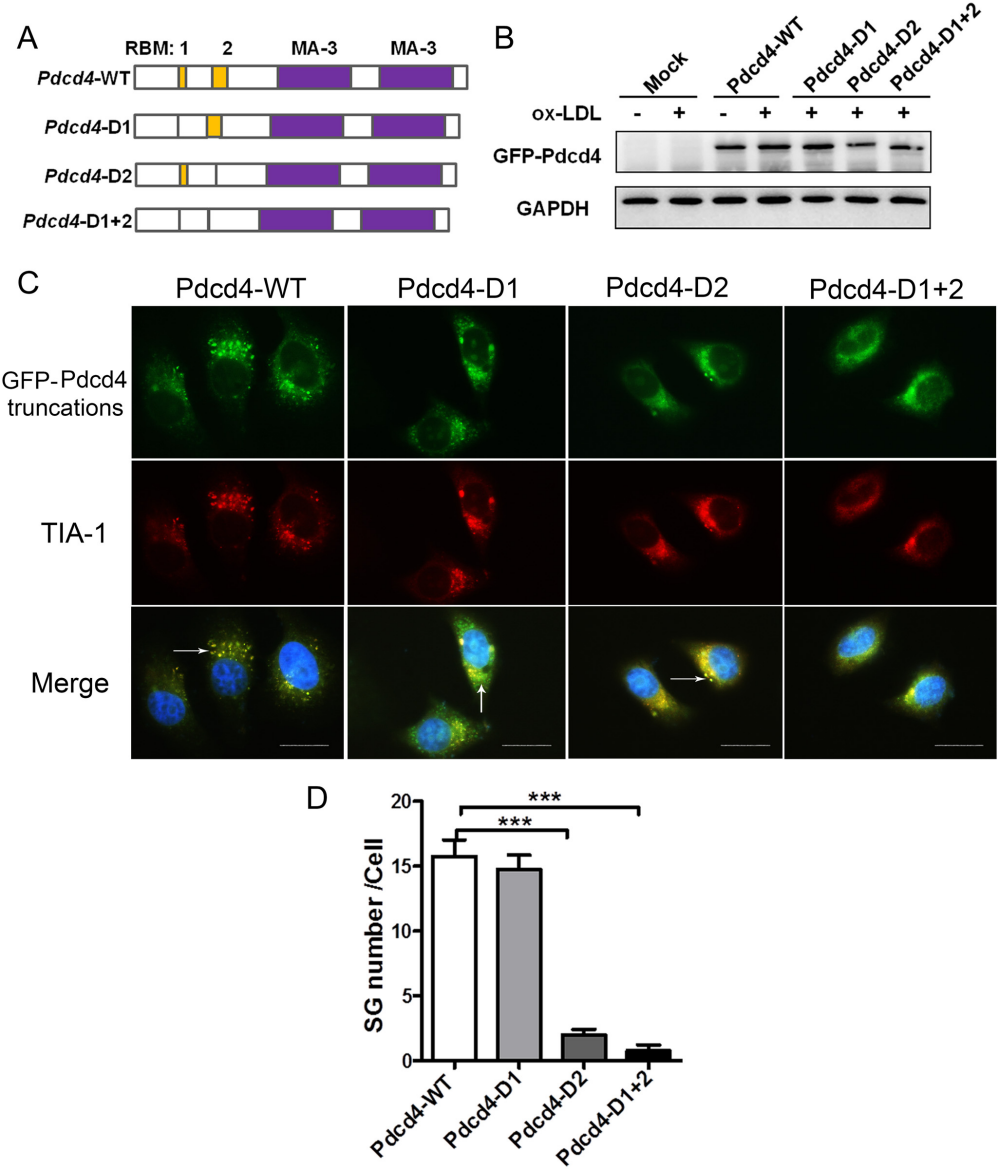
Pdcd4 participates in the SG formation through its RNA-binding region. (A) Structural models showing the location of two RNA-binding motifs (RBM1 and RBM2) in WT *Pdcd4* genes, and three different truncated *Pdcd4* genes depleted of RBM1, RBM2, or both, respectively. (B) Representative western-blot for ectopic expression of GFP-Pdcd4 in HepG2 cells transfected with various truncated plasmids. (C) HepG2 cells were transfected with various truncated plasmids and then were stimulated with ox-LDL (50 μg/ml) for 16 h, the SG formation was examined by immunofluorescence. Exogenous Pdcd4 is visualized with GFP (green), TIA-1 was stained with rhodamine (red). Cells were counterstained with DAPI (blue). Arrow head indicates SGs in cells. The original magnification is 1000. Scale bar = 20μm. Data are from three independent experiments. (D) Number of SGs in HepG2 cells transfected with various truncated plasmids. Data are presented as mean ± s.e.m. ***P <0.001.

It has been recognized that two important regions, referred to as RBM1 and RBM2, within the N-terminal domain of Pdcd4 are involved in RNA-binding activity [29]. To determine whether these motifs affect the SG formation, based on *pEGFP-C1-Pdcd4 (Pdcd4-WT)* plasmid we constructed different truncated plasmids *pEGFP-C1-Pdcd4-D1* (*Pdcd4-D1*), *pEGFP-C1-Pdcd4-D2 (Pdcd4-D2)* and *pEGFP-C1-Pdcd4-D1+2 (Pdcd4-D1+2)*, which were depleted of RBM1, RBM2, both RBM1 and RBM2, respectively (Fig 5A). After transfection into HepG2 cells, ectopic GFP-Pdcd4 was detected in all truncate groups in response to ox-LDL (Fig 5B). Lots of apparent GFP^+^ SGs, which exhibited good co-localization with TIA-1, were detected in Pdcd4-WT-overexpressed cells. In contrast, in cells expressing Pdcd4-D2 or Pdcd4-D1+2, the formation of GFP^+^ TIA-1^+^ SGs remarkably decreased, which were hardly detected. For cells expressing Pdcd4-D1, there was no significant decrease in the number of GFP^+^ TIA-1^+^ SGs (Fig 5C and 5D). In the absence of ox-LDL, few GFP^+^ TIA-1^+^ SGs were detected in cells transfected with different truncated plasmids (S2 Fig). These data indicate that Pdcd4 participates in the assembly of ox-LDL-induced SGs mainly through its RNA-binding region RBM2.

### AKT-eIF2α axis is involved in the regulation of Pdcd4 on ox-LDL-stimulated SGs

**Fig 6:**
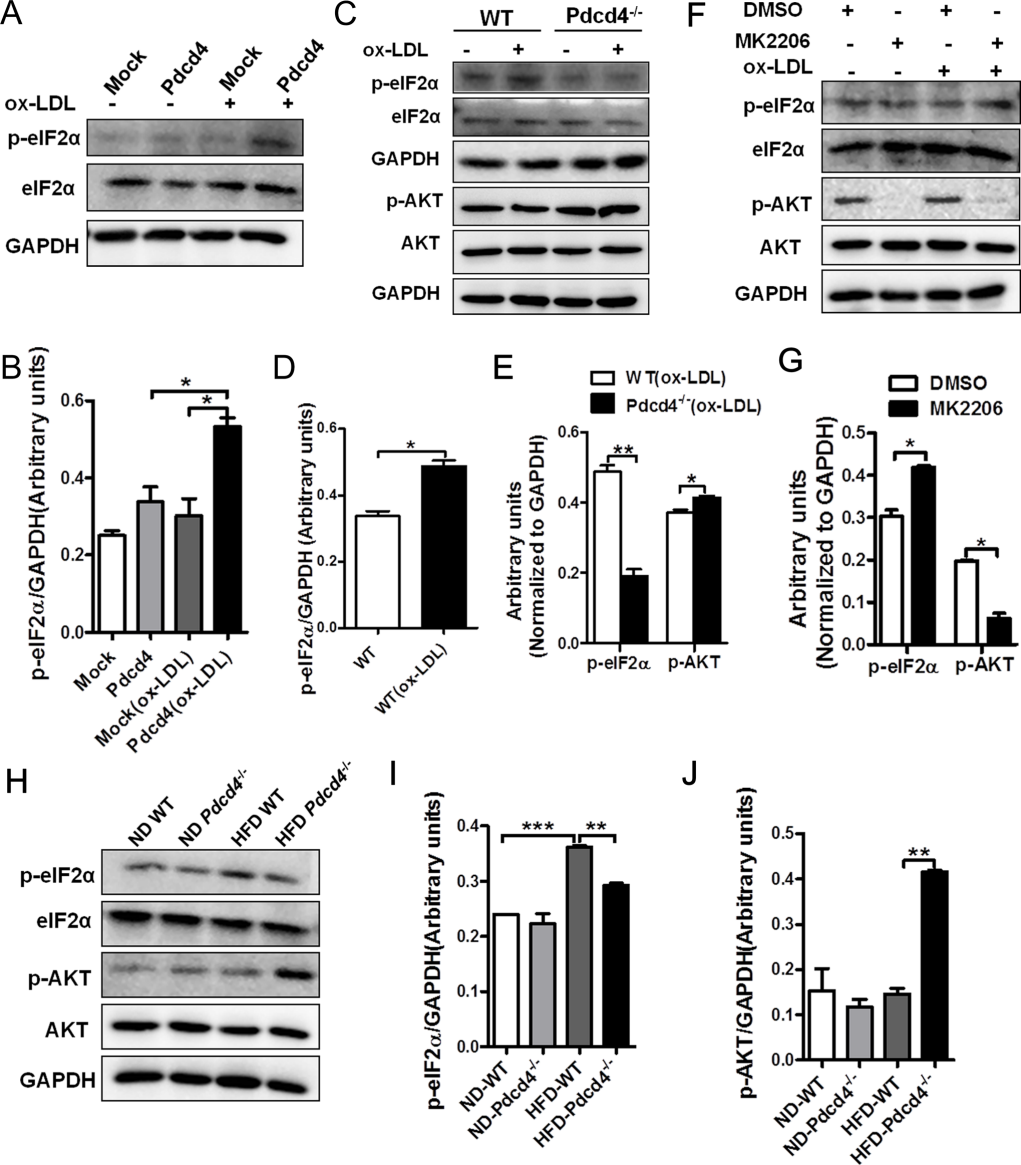
AKT-eIF2α axis is involved in the regulation of Pdcd4 on the formation of ox-LDL or HFD-induced SGs. (A, B) Western-blot assay of p-eIF2α in HeLa cell transfected with *pEGFP-C1* (Mock) or *pEGFP-C1-Pdcd4* (Pdcd4) in the presence or absence of ox-LDL (50 μg/ml). Representative (A) and statistic (B) data are shown. (C-E) Primary macrophages from WT and *Pdcd4*^-/-^ mice (n≥4 per group) were treated with ox-LDL (50 μg/ml) for 24 h. Representative western-blot (C) and statistic data of p-eIF2α and p-AKT (D, E) are shown. (F, G) Primary macrophages from *Pdcd4*^-/-^ mice (n = 4–6) were treated with ox-LDL (50 μg/ml) for 24 h in the presence or absence of MK2206 (2 μM). The expression of p-eIF2α and p-AKT were determined by western-blot. (H-J) Representative (H) and statistic (I, J) western-blot analysis of p-eIF2α and p-AKT in macrophages from WT and *Pdcd4*^-/-^ mice (n = 4 per group) fed on ND or HFD. Data are presented as mean ± s.e.m. *P <0.05, **P <0.01, ***P <0.001.

As eIF2α phosphorylation plays a pivotal role in the formation of SGs [2], we further examined the expression of p-eIF2α in HeLa cells. Fig 6A and 6B showed that ox-LDL treatment significantly enhanced the expression of p-eIF2α in Pdcd4-overexpressed HeLa cells, indicating that ox-LDL triggers the activation of eIF2α which contributes to the SG formation. Similarly, upon ox-LDL stimulation, a marked elevation in p-eIF2α levels was detected in WT macrophages but not in *Pdcd4*^*-/-*^ macrophages compared with non-treated ones (Fig 6C and 6D). Recently, Mounir et al reported the negative effect of AKT signaling on eIF2α phosphorylation which might contribute to relieved ER or oxidative stress [10]. To investigate the possible roles of AKT pathway in eIF2α phosphorylation and SG formation, we further detected the activation status of AKT in ox-LDL-treated WT and *Pdcd4*^*-/-*^ macrophages. In response to ox-LDL, *Pdcd4*^*-/-*^ macrophages displayed a moderate but significant elevation in AKT phosphorylation as compared to WT macrophages, which was consistent with the decline in eIF2α phosphorylation (Fig 6C and 6E). These results hint the reduction of p-eIF2α in *Pdcd4*^*-/-*^ macrophages in response to ox-LDL might be caused by the activation of AKT. To confirm this hypothesis, we further blocked AKT activation using an inhibitor MK2206 in ox-LDL-stimulated *Pdcd4*^*-/-*^ macrophages. As expected, after blocking AKT phosphorylation, the expression of p-eIF2α substantially reversed to a relatively higher level (Fig 6F and 6G), accompanied by an obvious increase of SG formation (S3 Fig). On the other hand, knockdown of AKT in *Pdcd4*^*-/-*^ macrophages using AKT siRNA, also led to increased level of p-eIF2α and SG formation in response to ox-LDL (S4 Fig). In addition, results from WT and *Pdcd4*^*-/-*^ mice fed on HFD also supported the changing trend of AKT-eIF2α axis in Pdcd4-associated SGs. Obvious reduction of eIF2α phosphorylation accompanied by enhancement of AKT activation were detected in macrophages from HFD-fed *Pdcd4*^*-/-*^ mice compared with those from HFD-fed obese WT mice (Fig 6H–6J). These findings indicate the potential regulatory role of AKT pathway in Pdcd4-involved SG formation through p-eIF2α.

## Discussion

SG assembly is a highly complex process during the cells response to adverse environmental conditions. Transient SGs appear to have cytoprotective effects that influence cell metabolism and survival [1, 2, 30], whereas prolonged SG activity is predicted to lead to a persistent stressed state and sustained translational repression that is deleterious [31, 32]. Sustained SG assembly has been recognized as a conspicuous feature of some degenerative diseases such as amyotrophic lateral sclerosis and Alzheimer's disease [31, 33, 34]. In the present study, we showed that ox-LDL, a biomarker of persistent oxidative stress in obesity and related metabolic syndromes [20, 21], induced obvious SG formation in WT macrophages. In HFD-fed obese mice, numerous SGs were also detected in macrophages and fatty liver tissues. However, compared with ox-LDL, HFD induces relatively few SGs in macrophages (Figs 1B and 2C), possibly because of its indirect effects on macrophages or the influence by in vitro manipulation in the absence of HFD. Since ox-LDL is not only the product of oxidative stress, but also enhances oxidative stress by promoting the generation of reactive oxygen species, it is very likely that HFD-induced SGs are caused by ox-LDL-related oxidative stress. These observations reveal the involvement of SGs in obesity and related diseases, in which either HFD or ox-LDL brings long and chronic oxidative stress, thus contributing to sustained SG assembly. Therefore, SG assembly in obesity is not only a result of stress stimuli, but also indicates persistent unsolved oxidative or ER stress. On the other hand, sustained SGs in turn lead to persistent blockade of translation that may produce detrimental effects on cell survival and function. Although the precise roles of SGs need to be further investigated, it still raises the possibility that mitigating SG-associated stress responses in these pathological conditions.

Pdcd4 has been recognized as a translation repressor which inhibits translation in a cap-dependent way. Using its MA-3 domain, Pdcd4 binds to eIF4A and blocks the interaction of eIF4A and eIF4G, thus interrupting the formation of translation initiation complex [29]. These findings provide the possibility of Pdcd4 to be involved in SG assembly. The co-localization of Pdcd4 with SG markers like eIF4A, TIA-1 and FXR1 confirmed that Pdcd4 is a key component of ox-LDL-induced SGs. Of note, we have demonstrated that the RNA-binding activity of Pdcd4 plays a critical role during SG assembly. Depletion of RNA-binding motifs RBM2 or both RBM1 and RBM2 led to almost vanished SG formation in response to ox-LDL. Previous reports have verified the RNA-binding activity of Pdcd4 and the association of Pdcd4 with small ribosomal subunits [29]. These data provide some evidence and further strengthen the importance of RNA-binding by Pdcd4 in SG formation, suggesting that the involvement of Pdcd4 in SG assembly is dependent on RNA-binding activity.

As the central trigger of the integrated stress response, eIF2α phosphorylation on Ser51 reduces levels of the eIF2–GTP–tRNA_i_^Met^ ternary complex that is required for cap-dependent translation initiation, thereby leading to translational arrest, polysome disassembly, and SG assembly [1, 2, 30]. Our data showed markedly increased SG formation in WT macrophages stimulated with ox-LDL, accompanied by remarkable elevation in p-eIF2α levels, thus supporting that ox-LDL-induced SG formation is dependent on eIF2α phosphorylation. More interesting, Pdcd4 deficiency conferred macrophages resistance to SG formation in response to ox-LDL or HFD, meanwhile the levels of p-eIF2α dramatically decreased. Consistently, increased levels of p-eIF2α were detected in Pdcd4-overexpressed HeLa cells in response to ox-LDL, which simultaneously led to obvious SG assembly. These findings suggest that Pdcd4 could regulate SG formation induced by ox-LDL, and eIF2α may act as a regulatory hub during this process.

Since AKT activation has been demonstrated to inhibit eIF2α phosphorylation [10], to dissect the possible mechanisms for the suppression of p-eIF2α in *Pdcd4*^*-/-*^ macrophages, we further compared the expression levels of p-AKT in *Pdcd4*^*-/-*^ and WT macrophages. As expected, in response to ox-LDL or HFD, a significant increase of p-AKT levels together with a decrease of p-eIF2α levels were detected in *Pdcd4*^*-/-*^ macrophages compared with WT macrophages. Blockade of AKT activation in ox-LDL-stimulated *Pdcd4*^*-/-*^ macrophages led to significant increase in p-eIF2α level and SG formation. Therefore, as a sensor to stress signals ox-LDL or HFD, Pdcd4 promotes eIF2α phosphorylation and SG formation, at least partially by repressing AKT activation. There are also other possibilities that p-AKT regulates SG formation independent of eIF2α phosphorylation. One recent report has demonstrated that TORC2, which is involved in SG formation in Drosophila, is required for AKT phosphorylation on S505 and stability upon heat shock [9]. Although the phosphorylation of eIF2α is not required for SG formation in Drosophila during heat stress [4], whether and how AKT plays a role in SG formation in Drosophila remains to be investigated [9]. Notably, the possible regulatory roles of TORC2-AKT signaling in SG formation in Drosophila may not be applied to mammalian cells used in our study, because of the differences in species, stress stimulus, and phosphorylation at AKT residues. In addition, it has been reported that AKT can activate TORC1, which is involved in SGs [11, 35]. So, it is important to consider the role of TORC1 pathway when studying the regulation of p-AKT in SG formation. As for the regulation of Pdcd4 on AKT activity, it has been reported in several types of cells, but detailed mechanisms remain to be elucidated. One possible explanation is that low level of Pdcd4 de-represses the activity of eIF4A RNA helicase, which contributes to the activation of PI3K-AKT pathway by increasing the translation of eIF4A-sensitive mRNA [36–38].

**Fig 7:**
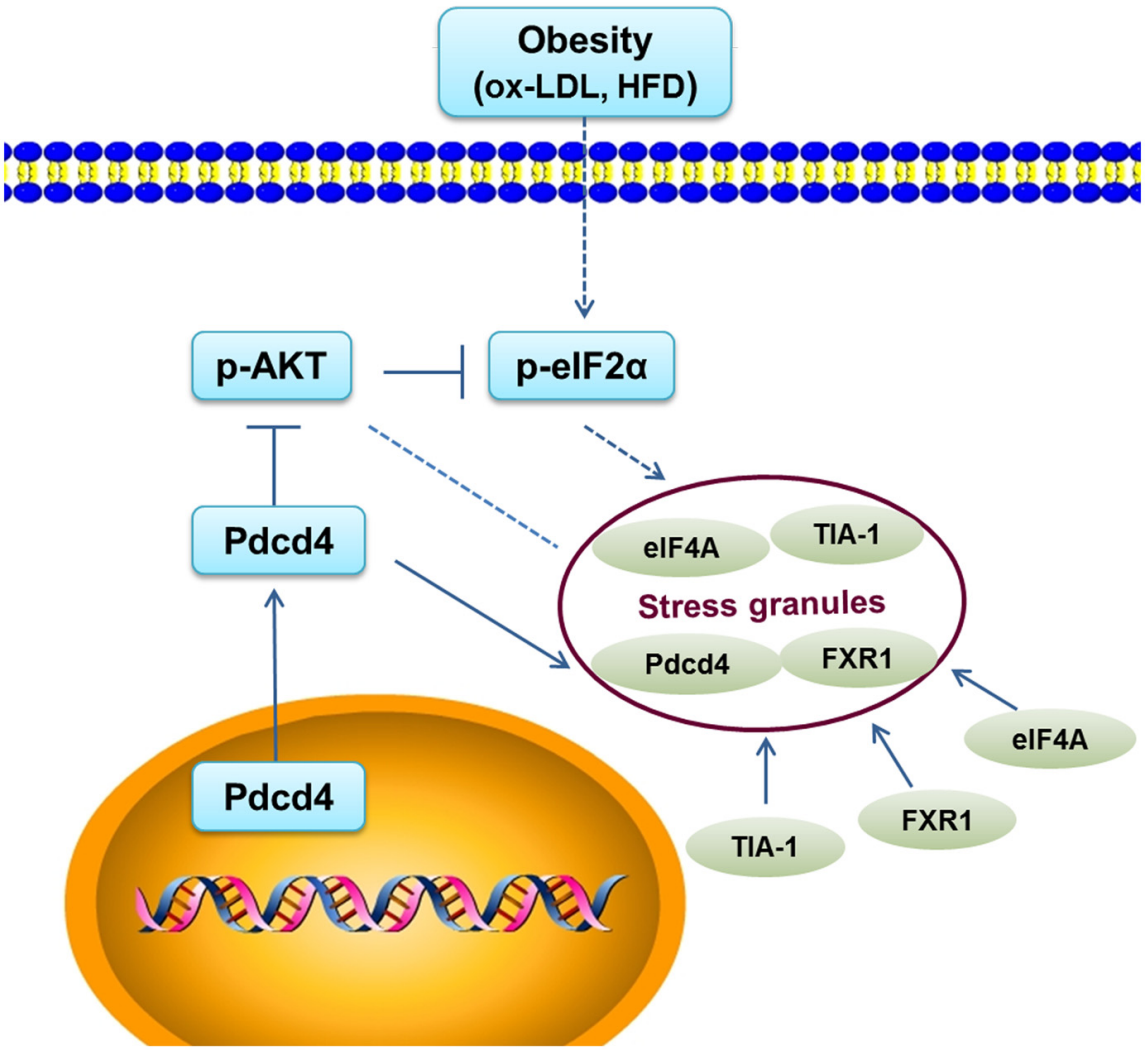
A working model shows the roles of Pdcd4 in the formation of SGs induced by ox-LDL or HFD. Pdcd4 serves as a key component of ox-LDL-induced SGs through its RNA-binding activity. On the other hand, Pdcd4 can enhance eIF2α phosphorylation by attenuating AKT activation, thereby promoting the formation of SGs.

Collectively, stress signals like ox-LDL or HFD have the ability to induce SG accompanied by eIF2α phosphorylation. This process is dependent on the involvement of Pdcd4, which not only functions as a key component of SG assembly via its RNA-binding region, but also promotes the phosphorylation of eIF2α by repressing AKT activation (Fig 7). Furthermore, it is also interesting to clarify the role of Pdcd4 in SG formation induced by other stimuli like heat shock or arsenite, which need to be further investigated. In conclusion, our data suggest that Pdcd4 plays an indispensible role in the pathogenesis of obesity and related diseases, thus may serve as a potential therapeutic target for these diseases.

## Supporting Information

S1 Fig Expression of Pdcd4 and SG markers in primary WT macrophages. The formation of TIA-1^+^ SGs was examined in primary macrophages from WT mice by immunofluorescence. Pdcd4 (green); TIA-1, FXR1, eIF4A (red); nuclei (blue). The original magnification is 1000. Scale bar = 10 μm.
(TIF)

S2 Fig SG formation in HepG2 cells transfected with truncated plasmids. HepG2 cells were transfected with various truncated plasmids, the SG formation was examined by immunofluorescence. Exogenous Pdcd4 is visualized with GFP (green), TIA-1 was detected with rhodamine (red). Cells were counterstained with DAPI (blue). The original magnification is 1000. Scale bar = 10μm.
(TIF)

S3 Fig SG formation in *Pdcd4*^-/-^ macrophages after blocking AKT activation. Primary macrophages from *Pdcd4*^-/-^ mice were treated with ox-LDL (50 μg/ml) for 24 h in the presence or absence of MK2206 (2 μM). The formation of TIA-1^+^ SGs was assayed by immunofluorescence. TIA-1 (red); nuclei (blue). The original magnification is 1000. Scale bar = 20μm.
(TIF)

S4 Fig Effect of AKT knockdown on p-eIF2α level in *Pdcd4*^*-/-*^ macrophages. *Pdcd4*^-/-^ macrophages were transfected with control or AKT siRNA (Sigma) for 36 h using GenePORTER^®^ 2 Transfection Reagent (Genlantis), and then were treated with ox-LDL (50 μg/ml) for additional 24 h. The effect of AKT siRNA on AKT expression and the influence of AKT pathway on the level of p-eIF2α were determined by western-blot. Representative (A, C) and statistic (B, D) data was shown. **P <0.01, ***P <0.001. (E) The influence of AKT pathway on the formation of TIA-1^+^ SGs was assayed by immunofluorescence. TIA-1 (red); nuclei (blue). The original magnification is 1000. Scale bar = 10μm.
(TIF)
