# Supplementary figures and images for: Pdcd4 Is Involved in the Formation of Stress Granule in Response to Oxidized Low-Density Lipoprotein or High-Fat Diet
Source: PLoS One. 2016 Jul 25;11(7):e0159568. doi: 10.1371/journal.pone.0159568 (PMC4959751; doi:10.1371/journal.pone.0159568)

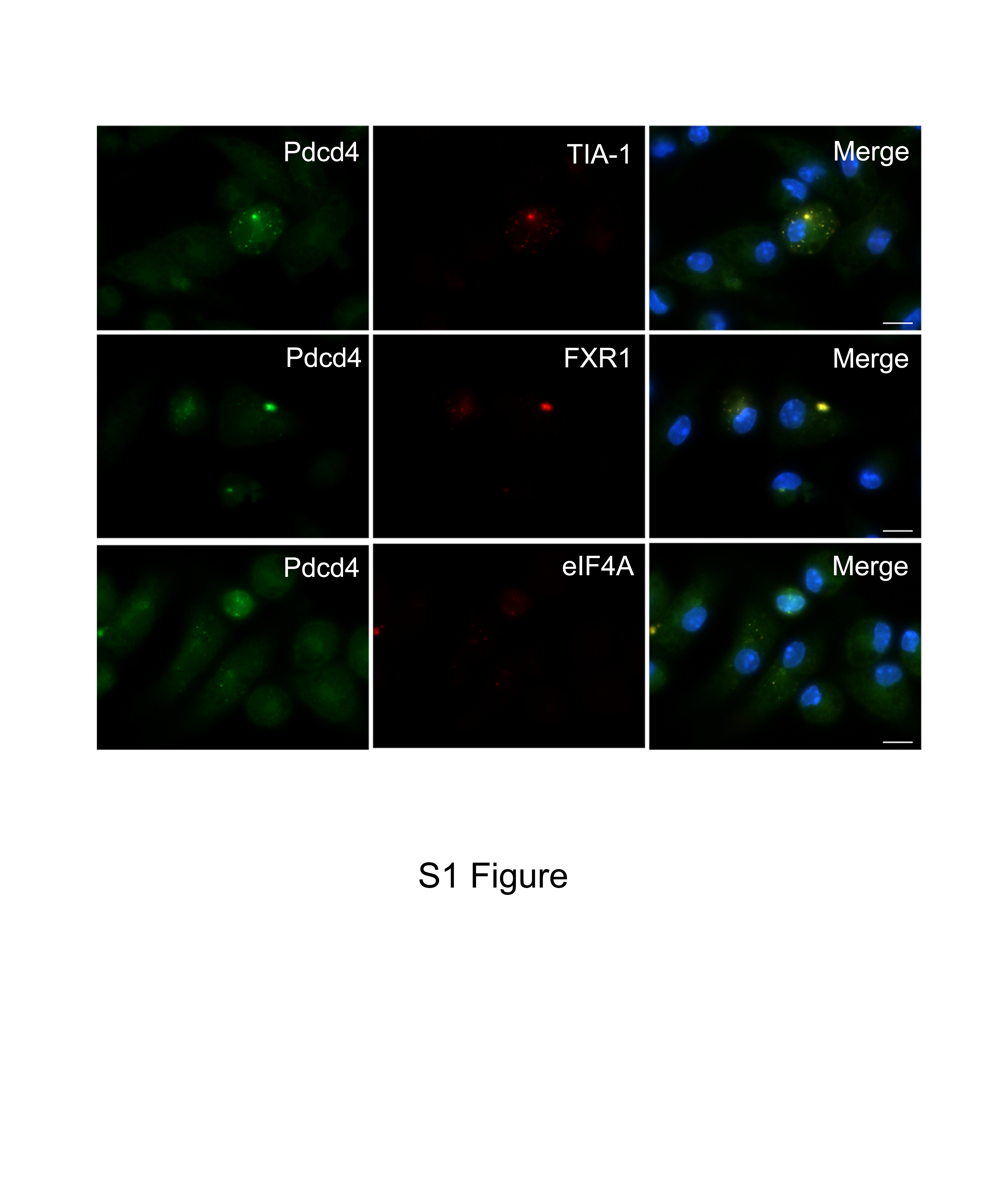

Supplement: S1 Fig — The formation of TIA-1+ SGs was examined in primary macrophages from WT mice by immunofluorescence. Pdcd4 (green); TIA-1, FXR1, eIF4A (red); nuclei (blue). The original magnification is 1000. Scale bar = 10 μm. (TIF) [file pone.0159568.s001.tif]

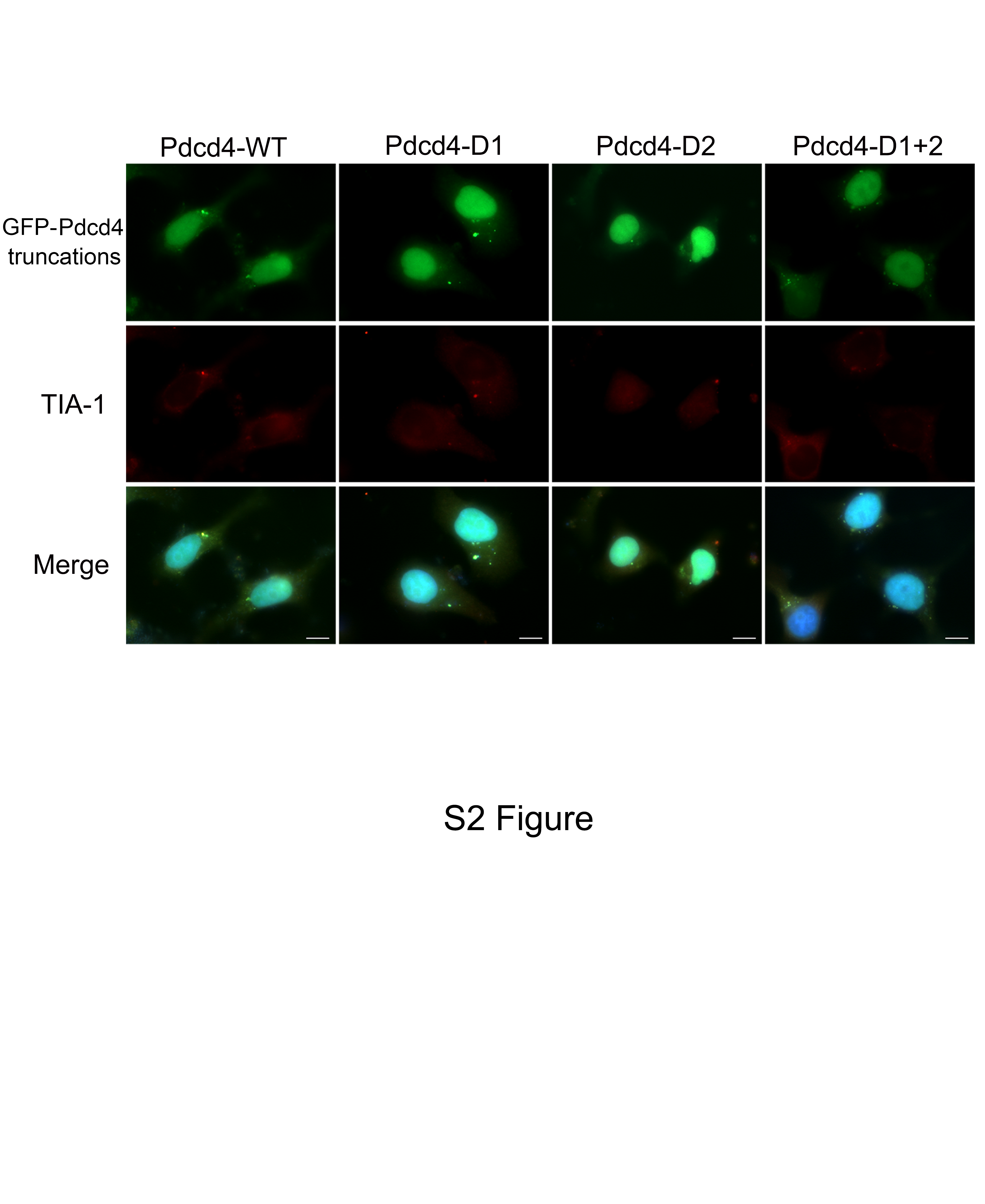

Supplement: S2 Fig — HepG2 cells were transfected with various truncated plasmids, the SG formation was examined by immunofluorescence. Exogenous Pdcd4 is visualized with GFP (green), TIA-1 was detected with rhodamine (red). Cells were counterstained with DAPI (blue). The original magnification is 1000. Scale bar = 10μm. (TIF) [file pone.0159568.s002.tif]

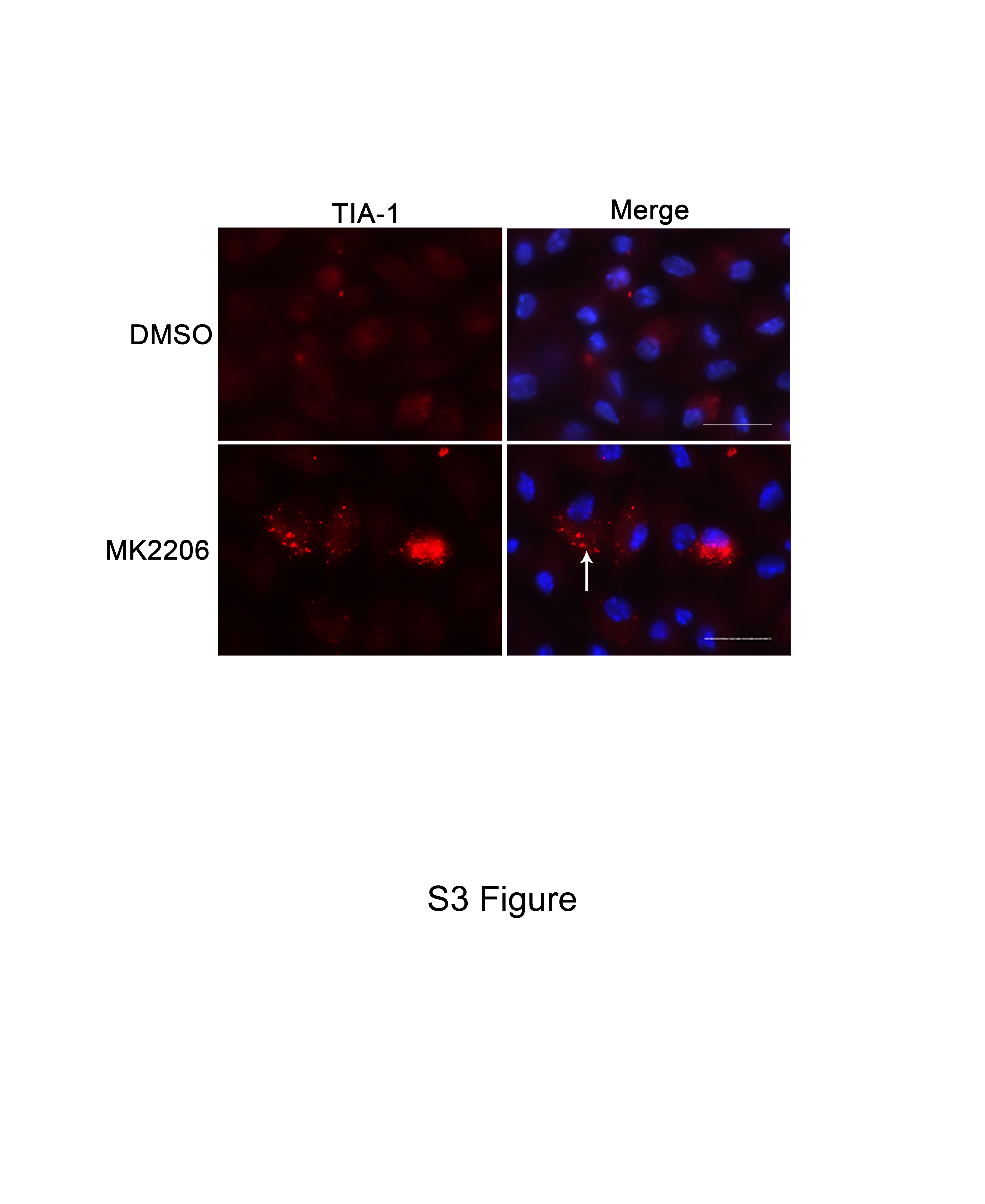

Supplement: S3 Fig — Primary macrophages from Pdcd4-/- mice were treated with ox-LDL (50 μg/ml) for 24 h in the presence or absence of MK2206 (2 μM). The formation of TIA-1+ SGs was assayed by immunofluorescence. TIA-1 (red); nuclei (blue). The original magnification is 1000. Scale bar = 20μm. (TIF) [file pone.0159568.s003.tif]

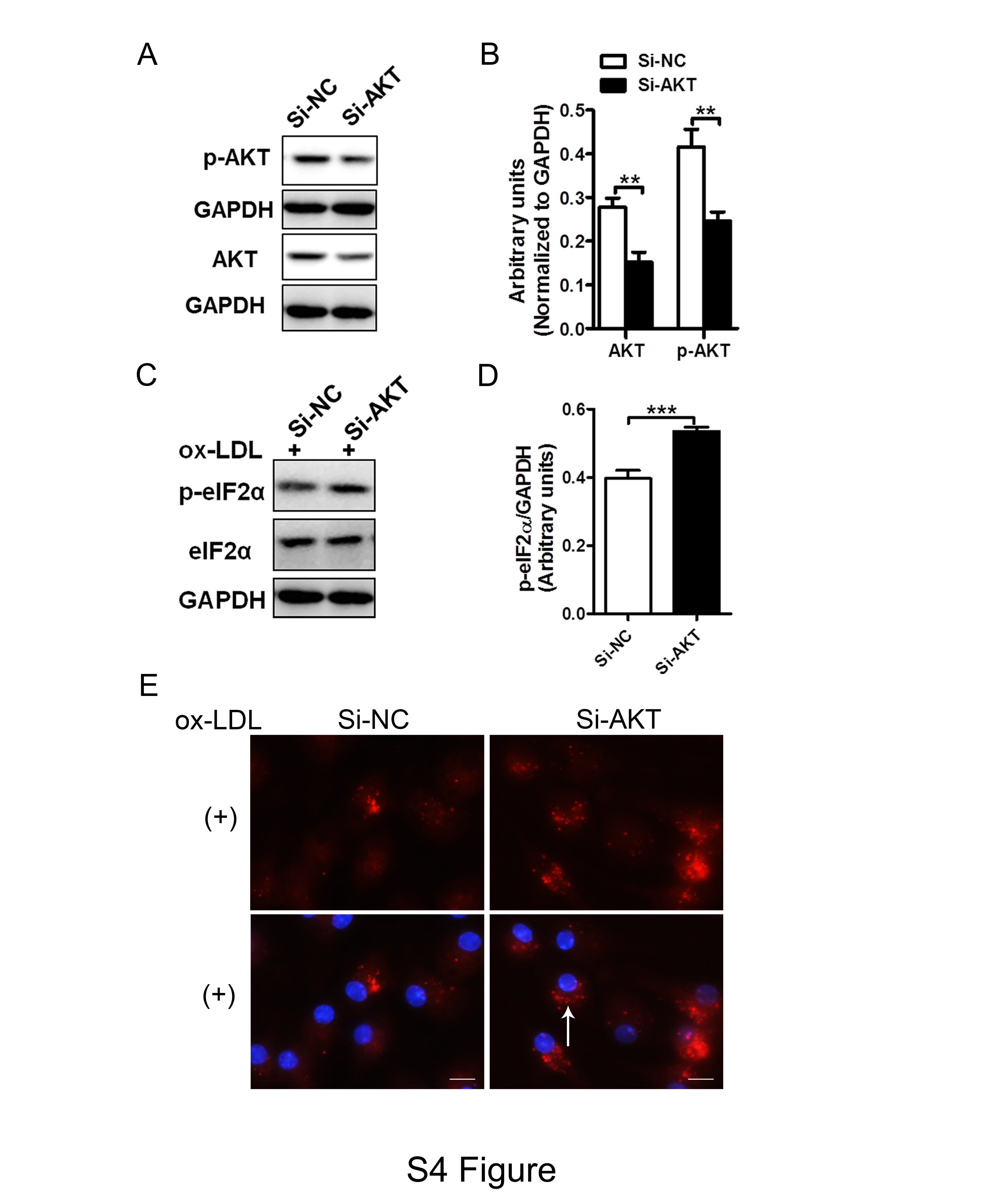

Supplement: S4 Fig — Pdcd4-/- macrophages were transfected with control or AKT siRNA (Sigma) for 36 h using GenePORTER® 2 Transfection Reagent (Genlantis), and then were treated with ox-LDL (50 μg/ml) for additional 24 h. The effect of AKT siRNA on AKT expression and the influence of AKT pathway on the level of p-eIF2α were determined by western-blot. Representative (A, C) and statistic (B, D) data was shown. **P <0.01, ***P <0.001. (E) The influence of AKT pathway on the formation of TIA-1+ SGs was assayed by immunofluorescence. TIA-1 (red); nuclei (blue). The original magnification is 1000. Scale bar = 10μm. (TIF) [file pone.0159568.s004.tif]
